# Use of nonsteroidal anti-inflammatory drugs and breast cancer risk in a prospective cohort of postmenopausal women

**DOI:** 10.1186/s13058-020-01343-1

**Published:** 2020-10-31

**Authors:** Manon Cairat, Marie Al Rahmoun, Marc J. Gunter, Gianluca Severi, Laure Dossus, Agnès Fournier

**Affiliations:** 1 Nutrition and Metabolism Section, International Agency for Research on Cancer, Lyon, France; 2 Centre de recherche en Epidémiologie et Santé des Populations (CESP), équipe “Exposome, Hérédité, Cancer et Santé”, Faculté de Médecine Université Paris-Saclay, UVSQ, Inserm U1018, Villejuif, France; 3 Gustave Roussy, Villejuif, France; 4 Department of Statistics, Computer Science and Applications “G. Parenti” (DISIA), University of Florence, Florence, Italy

**Keywords:** Nonsteroidal anti-inflammatory drugs, Breast cancer, Proton pump inhibitors, Postmenopausal women

## Abstract

**Background:**

Although anti-inflammatory agents could theoretically have anticancer properties, results from cohort studies on nonsteroidal anti-inflammatory drugs (NSAIDs) and breast cancer (BC) risk are inconsistent.

**Methods:**

We investigated the association between NSAID use and BC incidence in the French E3N prospective cohort, which includes 98,995 women born between 1925 and 1950 and insured by a health insurance plan that covers mostly teachers. Self-reported information on lifestyle and medical history has been collected biennially by questionnaires and matched with data from a drug reimbursement database covering the period 2004–2014. Women who self-reported current NSAID use in the 2000 or 2002 questionnaires or with at least two reimbursements in any previous 3-month period were defined as exposed to NSAIDs. Multivariable Cox regression models were used to estimate hazard ratios (HRs) for the association of NSAID use with BC risk.

**Results:**

In the current analysis, 62,512 postmenopausal women were followed between 2004 and 2014 (9 years on average, starting at a mean age of 63 years; 2864 incident BC). In multivariable models, there was no statistically significant association between NSAID use and BC risk [HR = 1.00 (0.92–1.08), compared with non-exposed women]. The NSAID-BC associations did not differ by NSAID types, BC subtypes, risk factors, and comorbidities, nor by duration and dose of use. However, a statistically significant interaction was observed by proton pump inhibitor (PPI) drug use (*P*_interaction_ = 0.01) whereby a decreased risk of BC with NSAID use was only observed among women who also used PPI before.

**Conclusion:**

Only women who used NSAIDs after having used PPI had a lower risk of BC. This result is novel and requires replication in other studies.

## Introduction

Breast cancer is, by far, the most common cancer among women in the world. In France, an estimated 56,162 new cancer cases have been diagnosed in 2018 and 13,353 women died from the disease [1]. Inflammation is recognized as a hallmark of cancer [2], and hence, it has been postulated that nonsteroidal anti-inflammatory drugs (NSAIDs) could have anticancer properties [3]. NSAIDs, including high-dose aspirin (pills of 500 mg or more), ibuprofen, or diclofenac, are commonly used to reduce inflammation associated with conditions such as arthritis, tendonitis, and bursitis, or to alleviate pain from osteoarthritis, headaches, migraine, muscle aches, toothaches, back pain, and menstrual cramps. All NSAIDs inhibit the cyclooxygenase (COX) enzymes 1 and 2 to different extents, leading to a reduction in prostaglandin biosynthesis [4], which could hypothetically promote apoptosis and inhibit mutagenesis [5, 6]. The latest meta-analysis of case-control and cohort studies suggests a slightly protective effect of NSAIDs (especially aspirin and selective COX-2 inhibitors) against invasive breast cancer, which seems to be restricted to hormone receptor-positive tumors [7]. However, authors detected a high degree of heterogeneity among the results from the different studies (especially across cohort studies) and highlighted that studies had limited data on subtypes of breast cancer, types of NSAIDs, and NSAID dose or duration of use. The inconsistencies across studies could be explained by the heterogeneous definitions of exposure (ever use, long-term use), the NSAID subtypes studied (all NSAIDs, all NSAIDs but aspirin, aspirin, ibuprofen, COX-2 inhibitors, etc.), or the exposure assessment period (past, current). In addition, over half of the prospective studies relied on self-reported data on NSAID use, prone to exposure misclassification, while studies exploring the associations between NSAID use and breast cancer risk using medico-administrative healthcare databases [8–17] had very limited data on potential confounding factors and were not able to evaluate whether the associations differed by specific breast cancer subtypes. Furthermore, there is a lack of epidemiological studies considering the use of other drugs as potential confounders/effect modifiers even though NSAIDs are frequently used concomitantly with other drugs such as proton pump inhibitors (PPIs) [18, 19].

We therefore investigated the association between NSAID use and breast cancer incidence, overall and by breast cancer subtypes, risk factors, and use of other drugs, in the French prospective E3N (Etude Epidémiologique auprès de femmes de la Mutuelle Générale de l’Education Nationale) cohort in which there are both self-reported information on lifestyle, medical, and reproductive factors, and drug reimbursement data for the period 2004–2014.

## Materials and methods

### E3N cohort

The E3N cohort includes 98,995 French women born between 1925 and 1950 and insured by the health insurance scheme that covers all workers in the national education system, most of whom are teachers [20]. In 1990, these volunteers provided their informed consent and completed the first questionnaire on their lifestyle, as well as lifetime medical and reproductive history. Every 2–3 years thereafter, participants completed self-administered questionnaires to mostly update this information and address the occurrence of medical events. Furthermore, for each cohort member, the health insurance plan provided data on all outpatient reimbursements for health expenditure since January 1, 2004. The study was approved by the French National Commission for Data Protection and Privacy.

### Identification of breast cancer cases

Most breast cancer cases were self-reported in the questionnaires and, to a lesser extent, identified from the clinical records from the patients or their doctors or the national cause-of-death registry. Pathology reports were obtained for 95% of the incident cases identified in the entire cohort and were used to confirm the cases and to extract information on tumor characteristics such as stage, grade, hormonal receptor status, and histological type. As the proportion of false-positive self-reports was low (< 5%), we did not exclude from our analyses cases for which we could not obtain pathology reports.

### Study population and follow-up

Since exposure was lagged by 6 months (see below), follow-up started on July 1, 2004. Participants contributed person-years of follow-up until the date of diagnosis of any cancer (with the exception of basal cell carcinoma and in situ colorectal tumor), the last completed questionnaire, or November 17, 2014 (date at which the last considered E3N questionnaire was sent to participants), whichever occurred first.

**Fig. 1:**
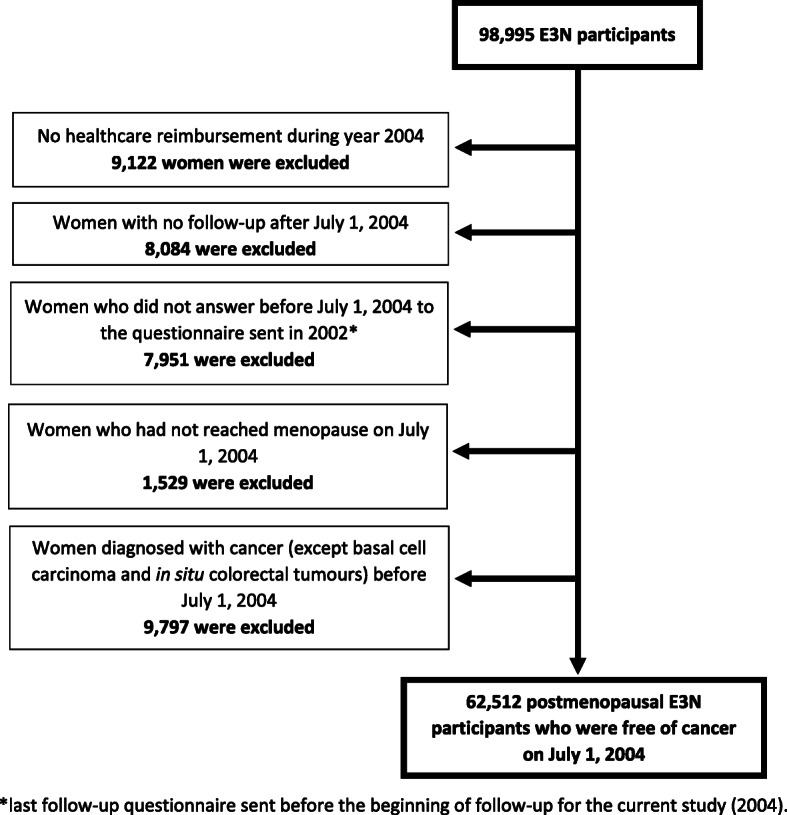
Flow chart

The study population included 62,512 postmenopausal women who were free of cancer on July 1, 2004 (Fig. 1).

### Exposure to NSAIDs

We considered all deliveries of NSAIDs since January 1, 2004 (as listed in Additional file 1, Table S1). Of note, aspirin used at low dose (pills of 325 mg or less) is not a NSAID but an antiplatelet drug.

The following data were extracted for each NSAID delivery: date of drug purchase, molecule, route of administration (oral, rectal, parenteral), number of pills/ampules per package, and dose per pill/ampule. E3N participants also reported whether they were currently using anti-inflammatory drugs at least 3 three times per week in the 2000 and 2002 questionnaires.

We defined as “NSAID ever users” women with at least one NSAID reimbursement since January 1, 2004, or who self-reported NSAID current use in the 2000 or 2002 questionnaires. We defined as “NSAID recurrent users” women with at least two reimbursements during any previous 3-month period since January 1, 2004, or who self-reported NSAID current use in the 2000 or 2002 questionnaires. Ever users who were not recurrent users were considered “occasional NSAID users.”

The types of NSAIDs that were the most frequently used in the cohort were individually analyzed: high-dose aspirin (≥ 500 mg), ibuprofen, ketoprofen, piroxicam, and diclofenac. Other NSAIDs were categorized according to their COX-selectivity [21]: selective COX-2 inhibitors (rofecoxib, etoricoxib, celecoxib), NSAIDs inhibiting preferentially COX-2 (meloxicam, etodolac, nimesulide), and other non-selective NSAIDs (mefenamic acid, indomethacin, tenoxicam, nabumetone, tiaprofenic acid, naproxen, fenoprofen, flurbiprofen, sulindac, phenylbutazone, aceclofenac, alminoprofen, niflumic acid, morniflumate, and floctafenine).

We also classified exposure among recurrent users according to characteristics of use: time since last use, time since first use, age at first use, cumulative duration of use, average daily dose use, and cumulative number of defined daily doses (DDD). The DDD is the assumed average daily maintenance dose for a molecule used for its main indication in adults, available from the WHO Collaborating Centre for Drug Statistics Methodology [22]. The DDDs are based on the treatment of pain, osteoarthritis, and rheumatoid arthritis, respectively, for aspirin, selective COX-2 inhibitors, and other NSAIDs. The duration of use was calculated as the shortest length of time between the standard duration of treatment contained in the box delivered and the time until the next NSAID delivery. We assumed that the standard duration was the number of pills/ampules per package for piroxicam, tenoxicam, and meloxicam or parenteral drugs. For other NSAIDs which might be taken several times a day, we assumed that the standard duration was the number of pills per package divided by 2. The cumulative duration of use was calculated as the sum of durations of use corresponding to each delivery since January 1, 2004. The date of last use was calculated as the date of last reimbursement + the standard duration of treatment contained in the last reimbursed box.

Women with NSAID deliveries between January 1, 2004, and April 1, 2004, were likely to have begun NSAID use before the availability of reimbursement data. Because the cumulative duration and dose, age at first use, and time since first use are in that case likely to be left-truncated, they were assigned to an “unknown” category, unless they could be assigned to a “> *x*” duration/dose/time since first/last use or a “< *x*” age at first use category.

### Covariates

Parameters considered as potential confounders are listed in Table 1. Some information on these parameters originated from the biennial self-administered questionnaires sent before year 2004 (educational level [1990], breastfeeding [1990 and 1992], age at menopause [up to 2004], age at menarche [1990], parity and age at first full-term pregnancies [1990 and 1992], current level of physical activity [2002], familial history of breast cancer [up to 2000], lifetime use of oral contraceptives [up to 2002]), with subsequent updates in 2005, 2008, and 2011 for most parameters that could change during follow-up (current body mass index [BMI], current smoking status, lifetime personal history of benign breast disease, current alcohol intake, self-report of a mammogram performed during the previous follow-up cycle, and lifetime histories of arthrosis, rheumatism, arthritis, polyarthritis, spondyloarthritis, or migraine). Number of consultations with the doctor during the preceding 6 months and drugs more likely to be used among NSAID users [paracetamol (Anatomical Therapeutic Chemical class (ATC, http://www.whocc.no/atc_ddd_index/) code: N02BE01), corticosteroids (H02AB), PPIs (A02BC), and symptomatic slow-acting drugs for osteoarthritis such as glucosamine (M01AX05), diacerein (M01AX21), oxaceprol (M01AX24), chondroitin sulfate (M01AX25), or avocado and soybean oil (M01AX26)] were identified using the drug reimbursement database which contains information starting from January 1, 2004. Lifetime use of menopausal hormone therapy (MHT) was identified using the drug reimbursement database as well as self-reported information from the questionnaires sent out before the year 2004 [23].

**Table 1:** Characteristics of participants, overall and according to NSAID exposure at the end of follow-up (E3N cohort, 2004–2014)

Characteristics at the end of follow-up^**1**^	All women (***n*** = 62,512)	NSAID exposure at the end of follow-up	
		Never/occasional users (***n*** = 24,019)	Recurrent users (***n*** = 38,493)
**Sociodemographic factors**			
**Age (years), mean (SD)**	72.1 (6.7)	71.8 (6.7)	72.2 (6.2)
**Educational level,** ***N*** **(%)**			
< High school	6516 (10)	2160 (9)	4356 (11)
From high school to 4 years higher education	45,138 (72)	17,450 (73)	27,688 (72)
At least 5 years higher education	10,858 (18)	4409 (18)	6449 (17)
**Lifestyle factors,** ***N*** **(%)**			
**BMI (kg/m**^**2**^**)**			
< 18.5	2626 (4)	1284 (5)	1342 (3)
[18.5–23[	25,409 (41)	10,913 (45)	14,496 (38)
[23–25[	13,197 (21)	5020 (21)	8177 (21)
[25–30[	16,139 (26)	5359 (22)	10,780 (28)
≥ 30	5141 (8)	1443 (6)	3698 (10)
**Physical activity (Met-h/week)**			
≤ 34.8	15,649 (25)	5863 (24)	9786 (25)
]34.8–57.6]	15,680 (25)	5986 (25)	9694 (25)
]57.6–88.8]	15,567 (25)	6085 (25)	9482 (25)
> 88.8	15,616 (25)	6085 (25)	9531 (25)
**Smoking status**			
Never smoker	33,281 (53)	13,258 (55)	20,023 (52)
Current smoker	4741 (8)	1681 (7)	3060 (8)
Past smoker	24,490 (39)	9080 (38)	15,410 (40)
**Alcohol intake (g/day)**			
Abstainer	7832 (13)	3283 (14)	4549 (12)
≤ 5	16,796 (27)	6611 (28)	10,185 (26)
]5–10]	9357 (15)	3752 (16)	5605 (15)
]10–20]	12,053 (19)	4546 (19)	7507 (20)
> 20	12,522 (20)	4483 (19)	8039 (21)
Missing	3952 (6)	1344 (6)	2608 (7)
**Reproductive factors**			
**Breastfeeding,** ***N*** **(%)**			
Never	23,425 (37)	9088 (38)	14,337 (37)
Ever	34,174 (55)	13,248 (55)	20,926 (54)
Missing	4913 (8)	1683 (7)	3230 (9)
**Age at menopause (years), mean (SD)**	50.52 (3.73)	50.68 (3.68)	50.41 (3.76)
**Age at menarche (years),** ***N*** **(%)**			
< 13	28,078 (45)	10,413 (43)	17,665 (46)
≥ 13	34,434 (55)	13,606 (57)	20,828 (54)
**Parity and age at first full-term pregnancy,** ***N*** **(%)**			
Nulliparous	7282 (12)	3010 (13)	4272 (11)
First child before age 30 years, one or two children	31,393 (50)	11,739 (49)	19,654 (51)
First child before age 30 years, three or more children	17,373 (28)	6602 (27)	10,771 (28)
First child after age 30 years	6464 (10)	2668 (11)	3796 (10)
**Ever use of oral contraceptives,** ***N*** **(%)**	38,570 (62)	14,191 (59)	24,379 (63)
**Lifetime MHT use,** ***N*** **(%)**			
Never	17,273 (28)	8076 (34)	9197 (24)
Recent	5300 (8)	1866 (8)	3434 (9)
Past	39,939 (64)	14,077 (59)	25,862 (67)
**Medical events and medical follow-up,** ***N*** **(%)**			
**Number of medical consultations/visits during the preceding 6 months**			
0	3277 (5)	1928 (8)	1349 (4)
[1–4[	25,413 (41)	11,387 (47)	14,026 (36)
≥ 4	33,491 (54)	10,434 (43)	23,057 (60)
Missing	331 (1)	270 (1)	61 (0)
**Self-report of a mammogram performed during the previous follow-up cycle**	51,097 (82)	19,194 (80)	31,903 (83)
**Personal history of benign breast disease**	23,268 (37)	8483 (35)	14,785 (38)
**History of breast cancer in first-degree relatives**	7139 (11)	2735 (11)	4404 (11)
**Recurrent use**^**2**^ **of other drugs,** ***N*** **(%)**			
**Systemic glucocorticoids**	17,391 (28)	3551 (15)	13,840 (36)
**Paracetamol**	38,392 (61)	10,527 (44)	27,865 (72)
**Proton pump inhibitors**	30,063 (48)	6308 (26)	23,755 (62)
**Anti-arthritics**	26,400 (42)	6471 (27)	19,929 (52)
**Comorbidities,** ***N*** **(%)**			
**History of arthrosis**	21,475 (34)	5568 (23)	15,907 (41)
**History of rheumatism**	6111 (10)	2400 (10)	3711 (10)
**History of arthritis**	888 (1)	248 (1)	640 (2)
**History of polyarthritis**	2375 (4)	451 (2)	1924 (5)
**History of spondyloarthritis**	236 (1)	29 (1)	207 (1)
**History of migraine**	21,591 (35)	7073 (29)	14,518 (38)

*Abbreviations*: *BMI* body mass index, *MET-h* metabolic equivalent task-hour, *MHT* menopausal hormone therapy, *NSAID* nonsteroidal anti-inflammatory drug, *SD* standard deviation

^1^Except for years of schooling, physical activity level, age at menarche, parity and age at first birth, lifetime use of oral contraceptives, history of breast cancer in first-degree relatives, and age at menopause, which were assessed before the start of follow-up

^2^At least two reimbursements during any previous 3-month period since January 1, 2004

### Statistical analysis

Multivariable Cox regression models with age as the time scale and stratified by birth cohort (in 5-year categories) were used to estimate hazard ratios (HRs) for the association of NSAID exposure with breast cancer incidence, overall and by breast cancer subtypes.

Exposure was considered as a time-varying parameter as well as other factors issued from the reimbursement database and covariates updated during follow-up. Consequently, participants classified as exposed to NSAIDs (either recurrent or occasional) or other drugs at the end of follow-up contributed follow-up as non-exposed until filling their first reimbursement (occasional) or their second reimbursement within a 3-month period (recurrent). Our time-varying approach also allowed us to update cumulative duration/dose and time since last and first use during follow-up. Undiagnosed breast cancer may cause symptoms resulting in an increased use of drugs a few months prior the diagnosis. A Danish study published in 2017 suggested that a 6-month lag time would be sufficient to minimize any reverse causation bias for most drug-cancer associations, including breast cancer [24]. Thus, exposure and other variables coming from the reimbursement database such as MHT, number of consultations, and other drugs were lagged by 6 months. Changing the lag time to 0, 1 year, or 2 years did not make a difference in the estimates (data not shown).

Educational level, recent mammogram (self-report of a mammogram performed during the previous follow-up cycle), and known risk factors for breast cancer were systematically included in the multivariable models [BMI, physical activity level, lifetime history of breast benign disease, family history of breast cancer, age at menarche, age at menopause, parity, lifetime use of oral contraceptives, lifetime use of MHT, and alcohol consumption]. Other lifestyle factors [ever breastfed, number of consultations with the doctor during the preceding 6 months, and smoking status] as well as comorbidities [lifetime histories of arthritis, arthrosis, polyarthritis, rheumatism, spondyloarthritis, and migraine] and recurrent use of selected drugs since January 1, 2004 [anti-arthritics, systemic glucocorticoids, PPI, and paracetamol], were tested as potential confounding factors. The categories used are displayed in Table 1. None of the additional covariates modified the HR by more than 0.05 points, and therefore, none was included in the final multivariable models. Analyses of each type of NSAIDs were mutually adjusted for use of other NSAID types.

Alcohol consumption had > 5% missing values, which were accommodated by using a “missing category.” All other covariates had < 5% of missing values, which were replaced either with the previous non-missing questionnaire value where appropriate, or with the mode or the median values observed among the subjects with complete data. A complete case analysis was also conducted (not shown because results were unchanged).

Effect modification by age, BMI, MHT use, comorbidities, and other drugs that might be associated with NSAID use (all considered as time-varying parameters) was evaluated by including cross-product interaction terms in the Cox models.

When studying the risk of different breast cancers characterized by their invasiveness, receptor status, and histological or stage/grade subtype, competing risk analysis was performed using the cause-specific hazards approach [25, 26]. Cases with missing information on a considered characteristic were excluded from the corresponding analyses.

We also performed several sensitivity analyses. First, we restricted the study sample to women with incident use by excluding women who were using NSAIDs before the beginning of the follow-up. Second, we defined “recurrent users” as women with at least two reimbursements during any previous 6-month period since January 1, 2004, or who self-reported NSAID current use in the 2000 or 2002 questionnaires. Third, we did not consider self-reported information about current NSAID use in the 2000 or 2002 questionnaires to define ever users or recurrent NSAID users. Fourth, we restricted the study sample to women who self-reported having had a mammogram performed in the previous follow-up cycle.

All tests of statistical significance were two-sided, and significance was set at the .05 level. We performed all analyses using SAS software, version 9.4 (SAS Institute Inc., Cary, NC).

## Results

The characteristics of the study population (*n* = 62,512) are presented in Table 1 and Table S2 (Additional file 1). At the end of follow-up, 56,031 women (90%) had been ever exposed to NSAIDs and 38,493 (62%) had been recurrently exposed. Among the recurrent NSAID users, 7% had been recurrently exposed to aspirin, 21% to ibuprofen, 20% to diclofenac, 17% to ketoprofen, 16% to piroxicam, 13% to selective COX-2 inhibitors, 9% to preferentially COX-2 inhibitors, and 27% to other non-selective NSAIDs during follow-up. A total of 12,897 women (21%) had been recurrently exposed to at least 2 categories of NSAIDs.

During a median follow-up time of 9 years, 2864 incident breast cancer cases were diagnosed (335 in situ and 2353 invasive). Compared to women who were occasionally or never exposed to NSAIDs, recurrent users were more often overweight or obese and had more frequent medical follow-up, exposure to other drugs (paracetamol, anti-arthritics, glucocorticoids, PPI, and MHT), and comorbidities such as migraine and arthrosis. There were no other major differences (Table 1).

**Fig. 2:**
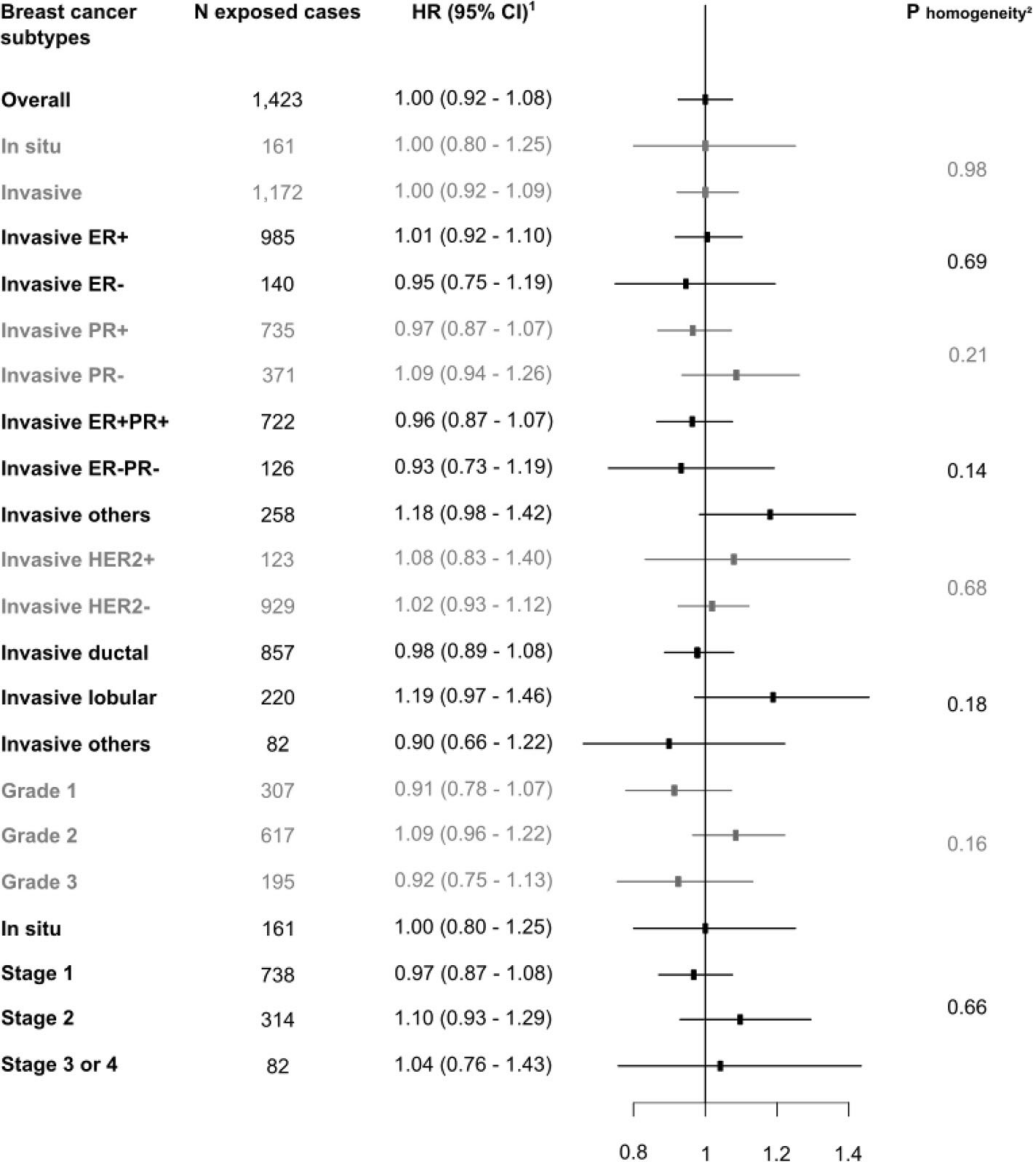
Associations of NSAID recurrent use with breast cancer risk, overall and by breast cancer subtypes, compared to never/occasional use (E3N cohort, 2004–2014). Abbreviations: CI, confidence interval; ER, estrogen receptor; HER2, human epidermal growth factor receptor 2; HR, hazard ratio; NSAID, nonsteroidal anti-inflammatory drug; PR, progesterone receptor. ^1^Adjusted for age (time scale), years of schooling (baseline), alcohol intake (time-varying), body mass index (time-varying), physical activity level (baseline), age at menarche (baseline), parity and age at first birth (baseline), lifetime use of oral contraceptives (baseline), age at menopause (baseline), history of breast cancer in first-degree relatives (baseline), personal history of benign breast disease (time-varying), lifetime use of menopausal hormone therapy (time-varying), and self-report of a mammogram performed during the previous follow-up cycle (time-varying). Categories used are those displayed in Table 1. ^2^Competing risk analysis was performed using the cause-specific hazards approach

The age-adjusted HR of breast cancer associated with having ever been exposed to NSAIDs, compared with having never been exposed, was 1.07 (95% CI, 0.98 to 1.18; Table 2). The multivariable HR was 1.02 (95% CI, 0.93 to 1.12). The multivariable HR of breast cancer associated with having been recurrently exposed to NSAIDs, compared with having been never or occasionally exposed, was 1.00 (95% CI, 0.92 to 1.08). No statistically significant heterogeneity across different types of NSAIDs (*P* homogeneity = 0.93; Table 2) and across breast cancer subtypes (*P* homogeneity between 0.14 and 0.98; Fig. 2) was found. Analyses according to characteristics of use yielded no statistically significant trend according to the number of DDDs, duration of use, average daily dose, age at first use, or time since first or last use (*P*_trend_ between 0.18 and 0.92; Table 3).

**Table 2:** Associations of NSAID exposure with breast cancer risk (E3N cohort, 2004–2014)

Characteristics of exposure	No. of cases	HR^**1**^ (95% CI)	HR^**2**^ (95% CI)
**Any NSAID**			
Never	596	1 (reference)	1 (reference)
Ever	2268	1.07 (0.98–1.18)	1.02 (0.93–1.12)
Never	596	1 (reference)	1 (reference)
Occasional	845	1.06 (0.95–1.18)	1.03 (0.92–1.15)
Recurrent	1423	1.09 (0.98–1.20)	1.01 (0.92–1.14)
Never/occasional	1441	1 (reference)	1 (reference)
Recurrent	1423	1.05 (0.97–1.13)	1.00 (0.92–1.08)
**Types of NSAID**			
**High-dose aspirin**			
Never/occasional use	2763	1 (reference)	1 (reference)
Recurrent use	101	1.06 (0.87–1.29)	1.02 (0.83–1.24)
**Ibuprofen**			
Never/occasional use	2659	1 (reference)	1 (reference)
Recurrent use	205	0.93 (0.81–1.08)	0.91 (0.79–1.05)
**Diclofenac**			
Never/occasional use	2650	1 (reference)	1 (reference)
Recurrent use	214	1.01 (0.88–1.17)	0.98 (0.85–1.14)
**Piroxicam**			
Never/occasional use	2651	1 (reference)	1 (reference)
Recurrent use	213	1.06 (0.92–1.22)	1.03 (0.90–1.19)
**Ketoprofen**			
Never/occasional use	2679	1 (reference)	1 (reference)
Recurrent use	185	1.06 (0.91–1.23)	1.02 (0.88–1.19)
**Selective COX-2 inhibitors**			
Never/occasional use	2694	1 (reference)	1 (reference)
Recurrent use	170	1.02 (0.87–1.20)	0.98 (0.84–1.15)
**Other NSAIDs inhibiting preferentially COX-2**			
Never/occasional use	2740	1 (reference)	1 (reference)
Recurrent use	124	1.04 (0.87–1.25)	1.03 (0.86–1.24)
**Other NSAIDs**			
Never/occasional use	2547	1 (reference)	1 (reference)
Recurrent use	317	1.07 (0.95–1.21)	1.04 (0.92–1.17)

*Abbreviations*: *CI* confidence interval, *COX* cyclooxygenase, *HR* hazard ratio, *NSAID* nonsteroidal anti-inflammatory drug

^1^Adjusted only for age (time scale)

^2^Adjusted for age (time scale), years of schooling (baseline), alcohol intake (time-varying), body mass index (time-varying), physical activity level (baseline), age at menarche (baseline), parity and age at first birth (baseline), lifetime use of oral contraceptives (baseline), age at menopause (baseline), history of breast cancer in first-degree relatives (baseline), personal history of benign breast disease (time-varying), lifetime use of menopausal hormone therapy (time-varying), self-report of a mammogram performed during the previous follow-up cycle (time-varying), and other types of NSAIDs (except for “Any NSAID”) (time-varying). Categories used are those displayed in Table 1

**Table 3:** Associations of NSAID recurrent use with breast cancer, according to characteristics of use (E3N cohort, 2004–2014)

Characteristics of exposure	No. of cases	HR^**1**^ (95% CI)	***P***_**trend**_^**2**^
**Cumulative number of DDDs**			***0.67***
Never/occasional use of NSAIDs	1441	1 (reference)	
≤ 200	763	1.02 (0.93–1.12)	
> 200 to ≤ 400	89	0.93 (0.75–1.16)	
> 400 to ≤ 600	22	1.28 (0.84–1.95)	
> 600	43	1.02 (0.75–1.39)	
Unknown	506	0.96 (0.91–1.13)	
**Cumulative duration of use, months**			***0.92***
Never/occasional use of NSAIDs	1441	1 (reference)	
≤ 3	516	1.00 (0.91–1.11)	
> 3 to ≤ 6	256	1.05 (0.91–1.20)	
> 6 to ≤ 12	89	1.02 (0.82–1.27)	
> 12	73	1.03 (0.81–1.31)	
Unknown	489	0.96 (0.87–1.07)	
**Time since first use, years**			***0.85***
Never/occasional use of NSAIDs	1441	1 (reference)	
≤ 4	491	1.00 (0.90–1.11)	
> 4 to ≤ 6	201	1.07 (0.92–1.25)	
> 4 to ≤ 8	138	1.04 (0.86–1.25)	
> 8	249	0.88 (0.76–1.02)	
Unknown	344	1.01 (0.90–1.14)	
**Age at first use, years**			***0.85***
Never/occasional use of NSAIDs	1441	1 (reference)	
≤ 60	461	0.97 (0.86–1.09)	
> 60 to ≤ 65	292	1.06 (0.93–1.22)	
> 65 to ≤ 70	215	1.03 (0.88–1.20)	
> 70	154	1.03 (0.85–1.24)	
Unknown	301	0.95 (0.83–1.09)	
**Time since last use, years**			***0.79***
Never/occasional use of NSAIDs	1441	1 (reference)	
Current use	845	1.01 (0.93–1.11)	
> Current use to ≤ 2	330	1.02 (0.91–1.16)	
> 2 to ≤ 5	103	0.99 (0.81–1.22)	
> 5	57	0.85 (0.65–1.11)	
Unknown	88	0.89 (0.72–1.11)	
**Average daily dose**			***0.18***
Never/occasional use of NSAIDs	1441	1 (reference)	
≤ 1 DDD	445	1.03 (0.92–1.14)	
> 1 to ≤ 1.5 DDD	587	1.02 (0.92–1.13)	
> 1.5 to ≤ 2 DDDs	200	0.96 (0.83–1.12)	
> 2 DDDs	55	0.95 (0.73–1.25)	
Unknown	136	0.90 (0.76–1.08)	

*Abbreviations*: *CI* confidence interval, *DDD* defined daily dose, *HR* hazard ratio, *NSAID* nonsteroidal anti-inflammatory drug

^1^Adjusted for age (time scale), years of schooling (baseline), alcohol intake (time-varying), body mass index (time-varying), physical activity level (baseline), age at menarche (baseline), parity and age at first birth (baseline), lifetime use of oral contraceptives (baseline), age at menopause (baseline), history of breast cancer in first-degree relatives (baseline), personal history of benign breast disease (time-varying), lifetime use of menopausal hormone therapy use (time-varying), and self-report of a mammogram performed during the previous follow-up cycle (time-varying). Categories used are those displayed in Table 1. HRs were obtained from separate models including one characteristic of exposure at a time

^2^Tests for linear trends were performed among exposed women with known characteristics of exposure. The characteristics of use were considered as continuous variables

**Fig. 3:**
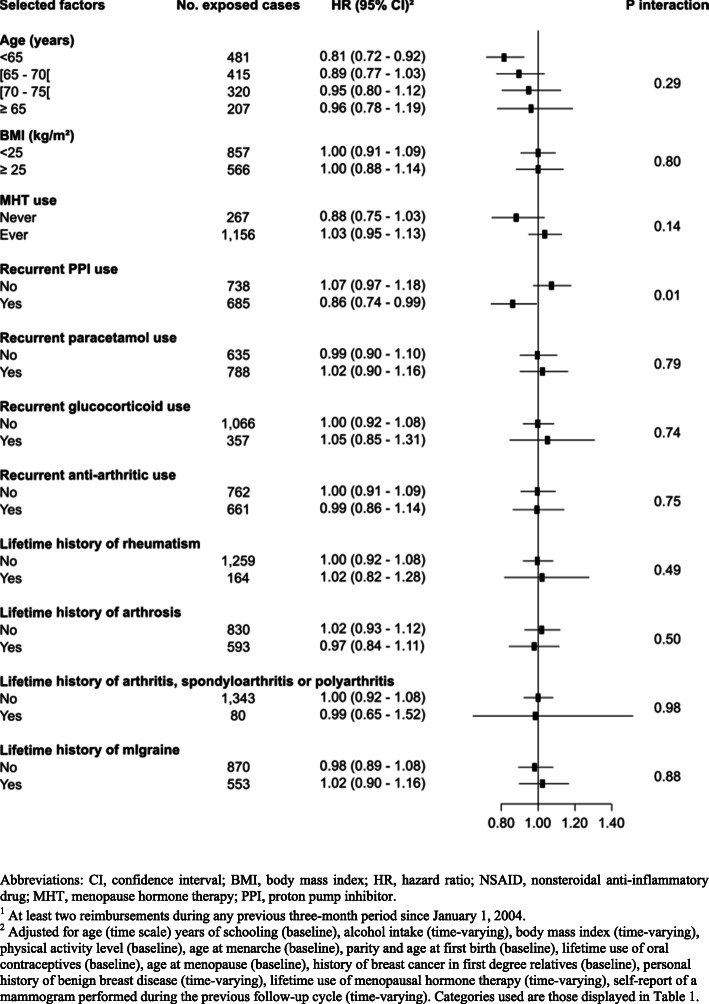
Associations of NSAID recurrent use with breast cancer risk, compared to never/occasional use, in strata of selected factors, comorbidities, and recurrent^1^ use of other drugs (E3N cohort, 2004–2014). Abbreviations: CI, confidence interval; BMI, body mass index; HR, hazard ratio; NSAID, nonsteroidal anti-inflammatory drug; MHT, menopause hormone therapy; PPI, proton pump inhibitor. ^1^At least two reimbursements during any previous 3-month period since January 1, 2004. ^2^Adjusted for age (time scale) years of schooling (baseline), alcohol intake (time-varying), body mass index (time-varying), physical activity level (baseline), age at menarche (baseline), parity and age at first birth (baseline), lifetime use of oral contraceptives (baseline), age at menopause (baseline), history of breast cancer in first-degree relatives (baseline), personal history of benign breast disease (time-varying), lifetime use of menopausal hormone therapy (time-varying), and self-report of a mammogram performed during the previous follow-up cycle (time-varying). Categories used are those displayed in Table 1

No effect modification by attained age, BMI, ever use of MHT, comorbidities, recurrent use of anti-arthritics, corticosteroids, and paracetamol was found (*P*_interaction_ > 0.14) (Fig. 3). However, there was a statistically significant interaction by PPI use. Recurrent NSAID use was inversely associated with overall breast cancer risk among women who had ever recurrently used PPIs [HR = 0.86 (0.74–0.99)] but not among never/occasional PPI users [HR = 1.07 (0.97–1.18); *P*_interaction_ = 0.01]. Associations between recurrent NSAID use and the risk of different breast cancer subtypes in strata of PPI use are shown in Table S3 (Additional file 1). Compared with women never exposed to NSAIDs nor PPIs, concomitant use of NSAIDs and PPIs was not associated with breast cancer risk [HR = 1.00 (0.87–1.14); *n* exposed cases = 559], NSAID use after PPI use was inversely associated with breast cancer risk [HR = 0.81 (0.69–0.95); *n* exposed cases = 365], and PPI use after NSAID use was positively associated with breast cancer risk [HR = 1.13 (1.00–1.29); *n* exposed cases = 607]. Exclusive use of NSAID was not associated with breast cancer risk [HR = 1.05 (0.89–1.09); *n* exposed cases = 956], and exclusive use of PPI was not associated with breast cancer risk [HR = 0.99 (0.97–1.14); *n* exposed cases = 773] (*P* homogeneity = 0.03).

The results remained unchanged after restricting the study sample to women with incident use by excluding women who were using NSAIDs before the beginning of the follow-up, changing the definition of recurrent NSAID use to “at least 2 reimbursements during a 6-month period,” and when we did not consider questionnaire data for NSAID use (data not shown). Results were also the same when the study sample was restricted to women with a recent mammogram (HR = 0.97 (0.90–1.05); *n* exposed cases = 1264, Additional file 1, Table S4).

## Discussion

Overall, recurrent NSAID use was not associated with breast cancer risk in this large, prospective cohort of 62,512 postmenopausal women with up to 10 years of follow-up. We found no duration- or dose-response relationship or differential effect across types of NSAIDs or breast cancer subtypes. However, we found a statistically significant decreased risk of breast cancer with NSAID use among women who had been previously exposed to PPIs.

Our results are consistent with the majority of results from prospective studies, suggesting no reduced risk of breast cancer with NSAID use whether the exposure considered was any type of NSAIDs [12, 27–29], high-dose aspirin [17, 27, 30], ibuprofen [27, 31, 32], COX-2 inhibitors [27, 30], or non-aspirin NSAIDs [17, 33–38]. However, some other studies have reported a reduced risk of breast cancer with NSAIDs (any type) [16, 32, 35, 39], high-dose aspirin [32], ibuprofen [16], COX-2 inhibitors [11, 14, 16, 39], or non-aspirin NSAIDs [29, 30, 40, 41] while a few other studies indicated an increased risk of breast cancer with NSAIDs (any type) [10, 31], high-dose aspirin [10], ibuprofen [10], or COX-2 inhibitors [13, 15].

Among a dozen prospective studies with data on duration or dose of use, a few observed a dose/duration response [16, 28, 32, 40]. In our study, the NSAID-breast cancer association did not differ according to duration or dose of use.

In some previous studies, the NSAID-breast cancer associations were limited to ER+ and/or PR+ breast cancer [33, 42]. In our study, we did not observe any significant heterogeneity according to breast cancer subtypes. The indication for using NSAIDs and the concomitant use of other drugs could also be confounding factors that few studies were able to take into account. Even though we did not know the clinical indications for NSAID use, we were able to adjust or stratify the NSAID-breast cancer associations on several comorbidities that are indications for NSAID use (arthritis, arthrosis, polyarthritis, rheumatism, spondyloarthritis, and migraine), and on use of other drugs (PPI, glucocorticoids, paracetamol, and anti-arthritics). Women exposed to NSAIDs had more comorbidities and were more exposed to other drugs, but we found no confounding or modifying effect of these parameters apart from the statistically significant interaction with recurrent PPI use. A lower risk of breast cancer was found when NSAID use followed PPI dispensation but not when NSAIDs were started at or before PPI dispensation.

Our study was the first one to evaluate the NSAID-breast cancer associations according to PPI use. PPIs, inhibitors of gastric acid secretion, are approved for the treatment or prevention of a broad range of related gastric conditions such as gastroesophageal reflux, gastric and duodenal ulcers, or NSAID-induced gastrointestinal complications [18, 43]. PPIs alter (increase or decrease depending on the molecule) the absorption of other drugs by increasing the intragastric pH and interfering with the metabolism of other drugs [44, 45]. Surprisingly, several reviews concluded that few studies were published about potential drug interactions between PPIs and NSAIDs [45, 46]. These studies focused on the concomitant use of these two drugs and showed no influence of PPIs on the pharmacokinetics of NSAIDs [45, 46]. However, PPI use might increase the risk of an imbalance in gut microbiota composition, even 8 weeks after PPI treatment [47, 48]. This change in microbiota composition has been shown to exacerbate NSAID side effects [49].

The main strengths of this study included its prospective design and the use of information from a drug reimbursement database to identify NSAID exposure, which avoids differential recall bias between cases and non-cases and minimizes exposure misclassification. Indeed, a French survey showed that among 250 French pregnant women, around 70% thought that high-dose aspirin and ibuprofen were not NSAIDs [50], and prescription analyses noted that among 184 prescriptions which contained at least one NSAID, 98% were obtained for acute symptoms and consequently for a short duration, contributing to recall bias [51]. In addition, the detailed NSAID reimbursement data allowed us to consider precise information on NSAID exposure (including molecules, duration, dose, time since last and time since first use). In our cohort, we found that exposure to NSAIDs was associated with a decreased risk of colorectal cancer (HR = 0.67 (0.54–0.82) for ever versus never exposed women; Additional file 1, Table S5), in close agreement with estimates from meta-analyses [52, 53], which provides support for the accuracy of our assessment of NSAID exposure. However, because NSAID is mostly used sporadically (as noted in a study from the French National Health Insurance Claims [54]), our statistical power remained limited to evaluate the NSAID-breast cancer associations for a long duration of use. In addition, the period of follow-up was inadequate to examine the NSAID-breast cancer associations in different windows of susceptibility such as postpartum or perimenopause [55, 56]. Since data on NSAID reimbursements were combined with self-reported data on lifestyle, reproductive, and medical factors, we were able to take into consideration potential confounders and to examine interactions of NSAID use with breast cancer risk factors, comorbidities, and other drugs. However, although we adjusted for many potential confounders, we cannot exclude residual confounding. For example, we could not take into account the severity of rheumatoid polyarthritis or other comorbidities. In addition, a high number of comparisons were evaluated and the significant interaction with recurrent PPI use may be a chance finding.

Drug reimbursement data combined with information on lifestyle, reproductive factors, and medical events are a great asset, but these data have some limitations. Exposure assessment based on reimbursements rather than intake leads to an obvious misclassification of NSAID use since, in France, NSAIDs can also be obtained over-the-counter. Indeed, several surveys in France noted that between 70 and 80% interviewed persons had already used NSAIDs by self-medication [57, 58]. Furthermore, we had no data regarding the compliance/adherence to the purchased treatment. However, we assumed that women who need NSAIDs chronically will go to the doctor to get reimbursed. We defined recurrent users as women with at least 2 reimbursements during any 3-month period which would suggest that they took the drug. Self-reported data on NSAID use, which therefore included NSAIDs purchased over-the-counter and captured only taken medications, were only available before year 2004 and consequently were used to assess prevalent use of NSAIDs.

## Conclusion

To conclude, in this large, prospective cohort of postmenopausal women with up to 10 years of follow-up, we observed a decreased risk of breast cancer with NSAID use only among women who previously used PPI. Our study is the first one to evaluate the modifying effect of PPI on the NSAID-breast cancer associations. Therefore, our finding of an interaction between NSAID and PPI use regarding the risk of breast cancer deserves replication in other settings.

## Supplementary information

**Additional file 1: Table S1.** ATC codes and COX-2 selectivity of NSAIDs. **Table S2.** Characteristics of participants, overall and according to NSAID exposure at the end of follow-up (E3N cohort, 2004-2014). **Table S3.** Associations of NSAID recurrent use with the risk of different breast cancer subtypes, compared with NSAID never/occasional use, in strata of PPI use (E3N cohort, 2004-2014). **Table S4.** Associations of NSAID recurrent use with breast cancer risk among women with a recent mammogram (E3N cohort, 2004-2014). **Table S5.** Associations of NSAID exposure with colorectal cancer risk (E3N cohort, 2004-2014).

## Data Availability

The data and computing code required to replicate the results reported in this paper are available upon duly motivated request by contacting Dr. Agnès Fournier.

## Abbreviations

BMI: Body mass index
CI: Confidence interval
COX: Cyclooxygenase
DDD: Defined daily dose
HR: Hazard ratio
MHT: Menopausal hormone therapy
NSAID: Nonsteroidal anti-inflammatory drug
PPI: Proton pump inhibitor
