# Supplementary material for: Use of nonsteroidal anti-inflammatory drugs and breast cancer risk in a prospective cohort of postmenopausal women
Source: Breast Cancer Res. 2020 Oct 31;22:118. doi: 10.1186/s13058-020-01343-1 (PMC7603705; doi:10.1186/s13058-020-01343-1)
Supplement: Supplementary file 1 — Additional file 1: Table S1. ATC codes and COX-2 selectivity of NSAIDs. Table S2. Characteristics of participants, overall and according to NSAID exposure at the end of follow-up (E3N cohort, 2004-2014). Table S3. Associations of NSAID recurrent use with the risk of different breast cancer subtypes, compared with NSAID never/occasional use, in strata of PPI use (E3N cohort, 2004-2014). Table S4. Associations of NSAID recurrent use with breast cancer risk among women with a recent mammogram (E3N cohort, 2004-2014). Table S5. Associations of NSAID exposure with colorectal cancer risk (E3N cohort, 2004-2014). [file 13058_2020_1343_MOESM1_ESM.docx]

Table S1. ATC codes and COX-2 selectivity of NSAIDs.

| **ATC codes** | **Molecules** | **COX-2 selectivity** |
| --- | --- | --- |
| **N02BA01** | High-dose Aspirin | Non selective |
| **N02BA71** | High-dose aspirin in combination with psycholeptics | Non selective |
| **M01AA01** | Phenylbutazone | Non selective |
| **M01AB01** | Indometacine | Non selective |
| **M01AB02** | Sulindac | Non selective |
| **M01AB16** | Aceclofenac | Non selective |
| **M01AB05**  **M01AB55** | Diclofenac  Diclofenac in combination with PPI | Non selective  Non selective |
| **M01AB08** | Etodolac | Preferentially selective |
| **M01AC01** | Piroxicam | Non selective |
| **M01AC02** | Tenoxicam | Non selective |
| **M01AC06** | Meloxicam | Preferentially selective |
| **M01AE01** | Ibuprofen | Non selective |
| **M01AE02** | Naproxen | Non selective |
| **M01AE03** | Ketoprofen | Non selective |
| **M01AE04** | Fenoprofen | Non selective |
| **M01AE09** | Flurbiprofen | Non selective |
| **M01AE11** | Tiaprofenic acid | Non selective |
| **M01AE16** | Alminoprofen | Non selective |
| **M01AH02** | Rofecoxib | COX-2 inhibitors |
| **M01AH05** | Etoricoxib | COX-2 inhibitors |
| **M01AH01** | Celecoxib | COX-2 inhibitors |
| **M01AG01** | Mefenamic acid | Non selective |
| **M01AX01** | Nabumetone | Non selective |
| **M01AX02** | Niflumic acid | Non selective |
| **M01AX17** | Nimesulide | Preferentially selective |
| **M01AX22** | Morniflumate | Non selective |
| **N02BG04** | Floctafenine | Non selective |

Abbreviations: ATC, Anatomical therapeutic chemical; COX, cyclooxygenase; NSAID, nonsteroidal anti-inflammatory drug; PPI, proton pump inhibitor.

Table S2. Characteristics of participants, overall and according to NSAID exposure at the end of follow-up (E3N cohort, 2004-2014).

|  | **NSAID exposure at the end of follow-up** | | | |
| --- | --- | --- | --- | --- |
| **Characteristics at the end of follow-up^1^** | **Never users**  **(n=5,943)** | | **Occasional users (n=18,076)** | **Recurrent users**  **(n=38,493)** |
| **Sociodemographic factors** | | | | |
| **Age (years), mean (SD)** | | 71.8 (7.4) | 71.8 (6.4) | 72.2 (6.2) |
|  | |  |  |  |
| **Educational level, N (%)** | |  |  |  |
| < High-school | | 605 (10) | 1,555 (9) | 4,356 (11) |
| From high-school to 4 years higher education | | 4,260 (72) | 13,190 (73) | 27,688 (72) |
| At least 5 years higher education | | 1,078 (18) | 3,331 (18) | 6,449 (17) |
|  | |  |  |  |
| **Lifestyle factors, N (%)** | | | | |
| **BMI (kg/m²)** |  | |  |  |
| < 18.5 | 380 (6) | | 904 (5) | 1342 (3) |
| [18.5 – 23[ | 2,705 (46) | | 8,208 (45) | 14,496 (38) |
| [23 - 25[ | 1,170 (20) | | 3,850 (21) | 8,177 (21) |
| [25 – 30[ | 1,293(22) | | 4,066 (22) | 10,780 (28) |
| ≥ 30 | 395 (7) | | 1,048 (6) | 3,698 (10) |
|  |  | |  |  |
| **Physical activity (Met-h/week)**  ≤ 34.8  ]34.8 - 57.6]  ]57.6 – 88.8]  > 88.8 | 1,479 (25)  1,385 (23)  1,519 (26)  1,560 (26) | | 4,384 (24)  4,601 (25)  4,566 (25)  4,525 (25) | 9,786 (25)  9,694 (25)  9,482 (25)  9,531 (25) |
|  |  | |  |  |
| **Smoking status** |  | |  |  |
| Never smoker | 3,324 (56) | | 9,934 (55) | 20,023 (52) |
| Current smoker | 399 (7) | | 1,282 (7) | 3,060 (8) |
| Past smoker | 2,220 (37) | | 6,860 (38) | 15,410 (40) |
|  |  | |  |  |
| **Alcohol intake (g/d)** |  | |  |  |
| Abstainer | 952 (16) | | 2,331 (13) | 4,549 (12) |
| ≤ 5 | 1,664 (28) | | 4,947 (27) | 10,185 (26) |
| ]5 - 10] | 898 (15) | | 2,854 (16) | 5,605 (15) |
| ]10 - 20] | 1,027 (17) | | 3,519 (19) | 7,507 (20) |
| > 20 | 1,030 (17) | | 3,453 (19) | 8,039 (21) |
| Missing | 372 (6) | | 972 (5) | 2,608 (7) |
|  |  | |  |  |
| **Reproductive factors** | | | | |
| **Breastfeeding, N (%)** |  | |  |  |
| Never | 2,369 (40) | | 6,719 (37) | 14,337 (37) |
| Ever | 3,191 (54) | | 10,057 (56) | 20,926 (55) |
| Missing | 383 (6) | | 1,300 (7) | 3,230 (9) |
|  |  | |  |  |
| **Age at menopause (years), mean (SD)** | 50.7 (3.8) | | 50.7 (3.6) | 50.4 (3.8) |
|  |  | |  |  |
| **Age at menarche (years), N (%)** |  | |  |  |
| < 13 | 2,536 (43) | | 7,877 (44) | 17,665 (46) |
| ≥ 13 | 3,407 (57) | | 10,199 (56) | 20,828 (54) |
|  |  | |  |  |
| **Parity and age at first full-term pregnancy, N (%)** |  | |  |  |
| Nulliparous | 892 (15) | | 2,118 (12) | 4,272 (11) |
| First child before age 30 years, one or two children | 2,726 (46) | | 9,013 (50) | 19,654 (51) |
| First child before age 30 years, three or more children | 1,653 (28) | | 4,949 (27) | 10,771 (28) |
| First child after age 30 years | 672 (11) | | 1,996 (11) | 3,796 (10) |
|  |  | |  |  |
| **Ever use of oral contraceptives, N (%)** | 3,106 (52) | | 11,085 (61) | 24,379 (63) |
|  |  | |  |  |
| **Lifetime MHT use, N (%)** |  | |  |  |
| Never | 2,503 (42) | | 5,573 (31) | 9,197 (24) |
| Recent | 512 (9) | | 1,354 (7) | 3,434 (9) |
| Past | 2,928 (49) | | 11,149 (62) | 25,862 (67) |
|  |  | |  |  |
| **Medical events & medical follow-up, N (%)** | | | | |
| **Number of medical consultations/visits**  **during the preceding 6 months** |  | |  |  |
| 0 | 654 (11) | | 1,274 (7) | 1,349 (4) |
| [1 – 4[ | 2,801 (47) | | 8,586 (48) | 14,026 (36) |
| ≥ 4 | 2,278 (38) | | 8,156 (45) | 23,057 (60) |
| Missing | 210 (4) | | 60 (0) | 61 (0) |
|  |  | |  |  |
| **Self-report of a mammogram performed during the previous follow-up cycle** | 4,415 (74) | | 14,779 (82) | 31,903 (83) |
|  |  | |  |  |
| **Personal history of benign breast disease** | 1,951 (33) | | 6,532 (36) | 14,785 (38) |
|  |  | |  |  |
| **History of breast cancer in first-degree relatives** | 695 (12) | | 2,040 (11) | 4,404 (11) |
|  |  | |  |  |
| **Recurrent use^2^ of other drugs, N (%)** | | | | |
| **Systemic glucocorticoids** | 525 (9) | | 3,026 (17) | 13,840 (36) |
| **Paracetamol** | 1,792 (30) | | 8,735 (48) | 27,865 (72) |
| **Proton Pump inhibitors** | 1,228 (21) | | 5,090 (28) | 23,768 (62) |
| **Anti-arthritics** | 1,119 (19) | | 5,352 (30) | 19,929 (52) |
|  |  | |  |  |
| **Comorbidities, N (%)** | | | | |
| **History of arthrosis** | 1,251 (21) | | 4,317 (24) | 15,907 (41) |
| **History of arthritis** | 67 (1) | | 181 (1) | 640 (2) |
| **History of polyarthritis** | 98 (2) | | 353 (2) | 1,924 (5) |
| **History of rheumatism** | 671 (11) | | 1,729 (10) | 3,711 (10) |
| **History of spondyloarthritis** | 5 (0) | | 24 (0) | 207 (1) |
| **History of migraine** | 1,598 (27) | | 5,475 (30) | 14,518 (38) |

Abbreviations: BMI, body mass index; MET-h, metabolic equivalent task-hour MHT, menopausal hormone therapy; NSAID, nonsteroidal anti-inflammatory drug; SD, standard deviation.

^1^ Except for years of schooling, physical activity level, age at menarche, parity and age at first birth, lifetime use of oral contraceptives, history of breast cancer in first degree relatives and age at menopause, which were assessed before the start of follow-up.

^2^ At least two reimbursements during any previous three-month period since January 1, 2004.

Table S3. Associations of NSAID recurrent use with the risk of different breast cancer subtypes, compared with NSAID never/occasional use, in strata of PPI use (E3N cohort, 2004-2014).

|  |  | **Never/occasional PPI use strata** |  | **Recurrent PPI use strata** | P _interaction_ |
| --- | --- | --- | --- | --- | --- |
|  | No. cases recurrently exposed to NSAIDs | HR*^1^* (95% CI) | No. cases recurrently exposed to NSAIDs | HR*^1^* (95% CI) |  |
| **Breast cancer subtypes** |  |  |  |  |  |
| Overall | 738 | 1.07 (0.97 – 1.18) | 685 | 0.86 (0.74 – 0.99) | 0.01 |
| In situ  Invasive | 85  614 | 1.06 (0.81 – 1.40)  1.08 (0.97 – 1.20) | 76  558 | 0.86 (0.56 – 1.32)  0.88 (0.75 – 1.03) | 0.45  0.03 |
| Invasive ER+  Invasive ER- | 507  78 | 1.08 (0.96 – 1.21)  1.00 (0.76 – 1.33) | 478  62 | 0.88 (0.74 – 1.05)  0.90 (0.56 – 1.45) | 0.04  0.74 |
| Invasive PR+  Invasive PR- | 366  209 | 1.02 (0.89 – 1.17)  1.18 (0.99 – 1.41) | 369  162 | 0.85 (0.70 – 1.03)  0.99 (0.73 – 1.35) | 0.09  0.38 |
| Invasive ER+PR+  Invasive ER-PR-  Invasive others | 358  69  148 | 1.02 (0.89 – 1.16)  0.97 (0.72 – 1.30)  1.33 (1.07 – 1.65) | 364  57  110 | 0.86 (0.70 – 1.04)  0.95 (0.57 – 1.57)  0.99 (0.68 – 1.43) | 0.10  0.96  0.21 |
| Invasive HER2+  Invasive HER2- | 67  473 | 1.19 (0.87 – 1.64)  1.09 (0.97 – 1.23) | 56  456 | 0.90 (0.54 - 1.51)  0.90 (0.75 – 1.07) | 0.50  0.04 |
| Invasive ductal  Invasive lobular  Invasive others | 462  99  4 | 1.09 (0.97 – 1.23)  1.04 (0.80 – 1.34)  1.04 (0.71 - 1.50) | 395  121  36 | 0.79 (0.65 - 0.94)  1.81 (1.14 – 2.86)  0.71 (0.39 – 1.28) | 0.01  0.03  0.30 |
| Grade1  Grade2  Grade3 | 163  320  103 | 0.97 (0.80 – 1.18)  1.19 (1.02 – 1.37)  1.00 (0.78 – 1.28) | 144  297  92 | 0.81 (0.60 – 1.10)  0.91 (0.72 – 1.13)  0.84 (0.57 – 1.25) | 0.06  0.06  0.58 |
| In situ  Stade 1  Stade 2  Stade 3 or 4 | 85  390  165  36 | 1.06 (0.81 – 1.40)  1.04 (0.91 – 1.18)  1.19 (0.97 – 1.45)  1.02 (0.67 – 1.55) | 76  558  149  46 | 0.86 (0.56 – 1.32)  0.87 (0.71 – 1.07)  0.93 (0.68 – 1.27)  0.88 (0.50 – 1.54) | 0.45  0.12  0.20  0.82 |

Abbreviations: CI, confidence interval; ER, estrogen receptor; HER2, human epidermal growth factor receptor 2; HR, hazard ratio; NSAID, nonsteroidal anti-inflammatory drug; PPI, proton pump inhibitors; PR, progesterone receptor.

^1^ Adjusted for age (time scale) years of schooling (baseline), alcohol intake (time-varying), body mass index (time-varying), physical activity level (baseline), age at menarche (baseline), parity and age at first birth (baseline), lifetime use of oral contraceptives (baseline), age at menopause (baseline), history of breast cancer in first degree relatives (baseline), personal history of benign breast disease (time-varying), lifetime use of menopausal hormone therapy (time-varying), self-report of a mammogram performed during the previous follow-up cycle (time-varying). Categories used are those displayed in Table 1.

Table S4. Associations of NSAID recurrent use with breast cancer risk among women with a recent mammogram (E3N cohort, 2004-2014).

| **Characteristic of Exposure** | **No. cases** | | **HR^1^ (95% CI)** |
| --- | --- | --- | --- |
| **Any NSAID** |  | |  |
| Never/occasional use  Recurrent use | 1,300  1,264 | | 1 (reference)  0.97 (0.90 – 1.05) |
| **Types of NSAID** |  | |  |
| **High-dose Aspirin**  Never/occasional use  Recurrent use | 2,472  92 | 1 (reference)  1.03 (0.83 – 1.26) | |
| **Ibuprofen**  Never/occasional use  Recurrent use | 2,384  180 | 1 (reference)  0.87 (0.75 – 1.02) | |
| **Diclofenac**  Never/occasional use  Recurrent use | 2,376  188 | 1 (reference)  0.97 (0.84 – 1.13) | |
| **Piroxicam**  Never/occasional use  Recurrent use | 2,368  196 | 1 (reference)  1.06 (0.92 – 1.23) | |
| **Ketoprofen**  Never/occasional use  Recurrent use | 2,392  172 | 1 (reference)  1.06 (0.91 – 1.24) | |
| **Selective COX-2 inhibitors**  Never/occasional use  Recurrent use | 2,418  146 | 1 (reference)  0.95 (0.80 – 1.13) | |
| **Other NSAIDs inhibiting preferentially COX-2**  Never/occasional use  Recurrent use | 2,450  114 | 1 (reference)  1.06 (0.88 – 1.28) | |
| **Other NSAIDs**  Never/occasional use  Recurrent use | 2,282  282 | 1 (reference)  1.02 (0.89 – 1.15) | |

Abbreviations: CI, confidence interval; COX, cyclooxygenase; HR, hazard ratio; NSAID, nonsteroidal anti-inflammatory drug.

^1^ Adjusted for age (time scale), years of schooling (baseline), alcohol intake (time-varying), body mass index (time-varying), physical activity level (baseline), age at menarche (baseline), parity and age at first birth (baseline), lifetime use of oral contraceptives (baseline), age at menopause (baseline), history of breast cancer in first degree relatives (baseline), personal history of benign breast disease (time-varying), lifetime use of menopausal hormone therapy (time-varying), self-report of a mammogram performed during the previous follow-up cycle (time-varying) and other types of NSAIDs (except for “Any NSAID”) (time-varying). Categories used are those displayed in Table 1.

Table S5. Associations of NSAID exposure with colorectal cancer risk (E3N cohort, 2004-2014).

| **NSAID exposure** | **No. cases** | **HR^1^ (95% CI)** | **HR² (95% CI)** |
| --- | --- | --- | --- |
| **Any NSAID** |  |  |  |
| Never | 134 | 1 (reference) | 1 (reference) |
| Ever | 356 | 0.66 (0.54 – 0.82) | 0.67 (0.54 – 0.82) |
|  |  |  |  |
| Never | 134 | 1 (reference) | 1 (reference) |
| Occasional | 134 | 0.68 (0.53 – 0.87) | 0.69 (0.54 – 0.88) |
| Recurrent | 222 | 0.65 (0.52 – 0.81) | 0.65 (0.52 – 0.82) |

Abbreviations: CI, confidence interval; HR, hazard ratio; NSAID, nonsteroidal anti-inflammatory drug.

^1^ Adjusted only for age (time scale).

² Adjusted for age (time scale), years of schooling (baseline), lifetime use of menopausal hormone therapy (time-varying), alcohol intake (time-varying), smoking status (time-varying), body mass index (time-varying) and physical activity level (baseline). Categories used are those displayed in Table 1.
